# Field Evaluation of Cholkit Rapid Diagnostic Test for *Vibrio Cholerae O1* During a Cholera Outbreak in Malawi, 2018

**DOI:** 10.1093/ofid/ofaa493

**Published:** 2020-10-16

**Authors:** Innocent Chibwe, Watipaso Kasambara, Mathews Kagoli, Harry Milala, Charity Gondwe, Andrew S Azman

**Affiliations:** 1 National Microbiology Reference Laboratory/Public Health Institute of Malawi, Malawi Ministry of Health, Lilongwe, Malawi; 2 Department of Epidemiology, Johns Hopkins Bloomberg School of Public Health, Baltimore, Maryland, USA

**Keywords:** cholera, malawi, rapid test, vibrio cholerae

## Abstract

Rapid diagnostic tests (RDTs) for cholera are an important emerging tool for surveillance, yet the currently available tests have several limitations. We assess the performance of a new RDT, Cholkit, during a cholera outbreak in Malawi compared with culture and find a sensitivity of 93.0% (95% CI, 83.0%–98.1%) and a specificity of 95.7% (95% CI, 78.1%–100.0%).

Cholera is an acute diarrheal infection caused by ingestion of *Vibrio cholerae.* Two serogroups, O1 and O139, are responsible for cholera epidemics. The current, seventh pandemic, which is responsible for the majority of cholera cases worldwide, is a *V. cholerae* O1 El Tor lineage. The O139 serogroup was first identified in South Asia in 1992 and has remained confined primarily to Asia, where its occurrence in humans is rare [1, 2].

Cholera confirmation relies on the identification of *V. cholerae* O1 or O139 by either stool culture or polymerase chain reaction (PCR) [3]. However, this procedure requires laboratory infrastructure, reagents, adequate transport procedures, and trained staff [4]. Rapid diagnostic tests (RDTs) represent a promising tool for early detection of *V. cholerae* O1 or O139 in areas without laboratory resources. They require less time than traditional methods like culture, minimum laboratory infrastructure, and basic technical skills [5].

While culture (followed by serologic confirmation) and PCR remain the gold standards for cholera identification and molecular characterization, RDTs can play a critical role in quickly identifying likely cholera outbreaks so that appropriate and timely public health responses can be initiated [6]. In addition, if the sensitivity and specificity of the tests are well defined, RDTs can serve as powerful surveillance tools to monitor trends in cholera over time and space.

There are currently 2 primary RDTs used for cholera globally, Crystal VC (Arkray Health Care Private Limited, Mumbai, Maharashtra, India) and SD Bioline (Standard Diagnostics Inc., Kyonggi province, Suwon city). Both of these immunochromographic tests are similar and rely on the detection of the lipopolysaccharide (LPS) antigen of *V cholerae* O1 or O139. While typically performed directly on stool, both tests have been shown to have improved specificity after samples are enriched in alkaline peptone water. Crystal VC is a bivalent O1/O139 test with sensitivity estimates ranging from 52.7%–97.0% when performed directly from stool to 62.6%–85.0% after enrichment [3, 7–9]. Specificity estimates for this test range from 49.2%–97.3% on direct stool to 91.2%–100% after enrichment (compared with culture) [3, 7–9]. Evaluations of other similar RDTs in Mozambique and Bangladesh have also shown similar performance [10, 11].

The O139 line on this test has been reported to give false positives [3, 12, 13] and given that O139 rarely circulates, its inclusion in the test may be more of a nuisance than an asset. The SD Bioline O1 test has demonstrated field sensitivity of 81.1%–90.9% and specificity of 92.8%–95.2% when performed directly, with increases in both after enrichment [14,15]. Both the Crystal VC and SD Bioline tests detect the lipopolysaccharides (LPS) antigens of *V. cholerae* using the precoated monoclonal antibodies specific to *V. cholerae* O1 and/or O139.

A new O1-only RDT, Cholkit, was recently developed in Bangladesh and is based on the detection of the O-specific polysaccharide component of the *V. cholerae* O1 LPS by monoclonal and colloidal gold particle–conjugated antibodies for detection of bound antigens. While a field study in Bangladesh has reported good test performance [8], there are no performance results published to date outside of this unique hyperendemic setting. Here we aim to assess the performance of the Cholkit Ag O1 RDT compared with a standard culture method for the confirmation of cholera cases during an outbreak in Malawi in addition to comparing it with a commonly used RDT.

## METHODS

This study was conducted in Nsanje and Chikwawa, districts in the Southern region of Malawi. The outbreak started after a flood disaster following a tropical disturbance over the Mozambique channel in March 2018, which left a trail of devastation in its wake. Heavy winds, widespread flooding, and landslides destroyed roads and bridges, farmlands and crops, and damaged houses, some beyond repair.

In total, 252 suspected cholera cases and 4 cholera-related deaths were reported in Malawi as part of this outbreak. Suspected cholera cases in this study were all individuals, regardless of age, presenting with acute diarrhea with moderate to severe dehydration in the cholera treatment camps. We attempted to enroll all suspected cases reporting to any of 5 cholera treatment centers between March and July 2018 in this study.

### Specimen Collection and Preparation

Fecal specimens (liquid stool or rectal swabs) were collected in a clean unchlorinated disposable container. The specimens were labeled and transported to the Nsanje District Hospital Laboratory within 2 hours of sample collection. If a >2-hour delay was expected, a stool-soaked swab was then placed into Cary-Blair transport medium and transported to the laboratory.

### Rapid Diagnostic Test Procedure

Once the stool sample arrived in the laboratory, cholera RDTs and culture were done directly from the sample. In addition, a stool-soaked swab from each sample was then inoculated in Alkaline Peptone Water (APW) for enrichment, which was kept between 4 and 6 hours at ambient temperature before RDT and culture, following the manufacturer’s instructions. Testing was performed by qualified and trained laboratory technologists from the Malawi National Microbiology Reference Laboratory. For Cholkit, 4 drops of watery stool were transferred into the sample processing vial (prefilled with 1 mL of sample diluent buffer), and the Cholkit strip was dipped into it for 15 minutes; the test line and/or control line appeared was red in color. Appearance of both lines indicated that the sample was positive for *V*. *cholerae* O1; appearance of only the control line but not the test line indicated a negative result for the test. Faint lines for both tests (O1 line for Crystal VC) were retested, and if they reappeared faint, they were classified as positive.

### Stool Culture

Samples were streaked on Thiosulphate Citrate Bile Sucrose Agar (TCBS) media after 4 hours of incubation in APW. After 18–24 hours of incubation at 37°C, TCBS plates were examined for the presence of yellow colonies suggestive of *V. cholerae*. Single-well isolated yellow colonies were picked and streaked on nonselective media (Nutrient agar) and incubated at 37°C for 24 hours. Colonies on nutrient agar were then tested for oxidase. If either the directly plated sample or enriched sample was positive, we considered the sample positive. Serotyping was conducted using polyvalent antisera on fresh isolates.

### Patient Consent

The study was done as part of the Public Health Institute of Malawi routine surveillance program. We did not seek written consent to test the stools of suspected cholera cases, as this study was considered exempt by the National Health Science Research Committee (NHSRC) of the Malawi Ministry of Health.

## RESULTS

Two hundred fifty-two suspected cholera cases were admitted to and treated in 5 health centers within the study area. Of these, 80 individuals had samples collected and their stool samples tested; none of these individuals reported antibiotic consumption before stool collection. The majority of samples tested, 49 (61.3%), were from participants who were severely dehydrated. All 80 samples were tested by culture, Cholkit, and Crystal VC.

In total, 57 (71.3%) samples were positive by culture (O1 Ogawa). When using the RDTs directly from stool, 53 (66.3%) were positive with Cholkit, with the same samples positive with Crystal VC (Table 1). After enrichment, 56 (70%) were positive with both Cholkit and Crystal VC (the same samples were positive in both tests). Nine direct tests had faint lines, 7 Crystal VC and 2 Cholkit after enrichment, with all 9 retesting as positive (and all 9 were culture positive). We had no O139 positives with the Crystal VC test. We found no discrepancies between Cholkit and Crystal VC. When used directly on stool, we found that the RDTs had a sensitivity of 93.0% (95% CI, 83.0%–98.1%) and a specificity of 95.7% (95% CI, 78.1%–100.0%) compared with culture. When tested after an enrichment step, we estimated a sensitivity of 98.2% (95% CI, 90.6%–100%) and a specificity of 100% (95% CI, 85.2%–100.0%).

**Table 1.** Overview of Cholkit Direct and Enriched Performance Results Compared With Culture

		Culture				
		Positive	Negative	Total	Sensitivity (95% CI), %	Specificity (95% CI), %
Direct RDT	Positive	53	1	54	93.0 (83.0–98.1)	95.7 (78.1–100.0)
	Negative	4	22	26		
	Total	57	23	80		
Enriched RDT	Positive	56	0	56	98.2 (90.6–100.0)	100.0 (85.2–100.0)
	Negative	1	23	24		
	Total	57	23	80		

## DISCUSSION

We found that during an outbreak in Malawi both Crystal VC and Cholkit RDTs had a high sensitivity and specificity for detecting *V. cholerae* compared with culture as a gold standard. The performance of both tests before and after enrichment exceeded the recommendations of minimal performance (90% sensitivity and 85% specificity) for RDTs proposed by the Global Taskforce for Cholera Control’s Laboratory Working Group [6, 16]. While Cholkit had the same sensitivity and specificity as Crystal VC, the combination of it being a simpler O1-only test and the reduced occurrence of faint lines may lead to reduced error in the field.

This study comes with a number of limitations. First, destruction of infrastructure, including road networks, by the floods complicated efforts to collect and transport specimens to the laboratory on time, with some samples arriving sometimes a full day after collection. Second, the specimen transfer method using a cotton swab may lack standardization in terms of the volume of specimen transferred into the sample transportation bottle, which could have affected the sensitivity of the assays. Third, our study population was more skewed toward severe suspected cases, which may have led to more sensitive results than in patients with less severe cholera (and hence lower bacterial concentration), thus limiting the generalizability of our results. Finally, while the testing was done in the region affected by the outbreak, no testing was done at the point of care by standard clinical staff. One previous study from the Democratic Republic of the Congo showed better RDT performance by trained laboratory technicians as opposed to clinicians, with no specific training on the use and interpretation of the RDTs [10, 13].

Our results are consistent with a previous study that evaluated the performance of Cholkit RDT in Bangladesh, which found a sensitivity of 98% and a specificity of 97% [17]. The performance of the Cholkit Ag O1 RDT suggests that this test could be used in the field to launch cholera alerts. While PCR and culture will likely remain the gold standard, RDTs like CholKit can be a useful tool for detecting cholera outbreaks early and tracking trends in the epidemiology of cholera.
